# Characterization of the complete plastid genome of *Acer tsinglingense*, an endemic tree species in China

**DOI:** 10.1080/23802359.2019.1689863

**Published:** 2019-11-18

**Authors:** Peng-Bin Dong, Yu Liu, Qi-Yuan Gao, Ting Yang, Xue-Yi Chen, Jia-Yu Yang, Qian-Han Shang, Min-Feng Fang

**Affiliations:** a Key Laboratory of Resource Biology and Biotechnology in Western China, Ministry of Education, College of Life Sciences, Northwest University, Xi’an, PR China;; b Department of Pharmacy, Traditional Chinese Medicine Hospital of Jingbian county, Jingbian, PR China;; c State Key Laboratory of Plateau Ecology and Agriculture, Qinghai University, Xining, PR China

**Keywords:** *Acer tsinglingense*, chloroplast genome, phylogenetic relationship

## Abstract

*Acer tsinglingense* is an ecologically and economically important tree species in China. In this study, we characterized its whole plastid genome sequence using the Illumina sequencing platform. The complete plastid genome size of *A. tsinglingense* is 156,039 bp in length, including a large single-copy [LSC] region of 85,760 bp, a small single-copy [SSC] region of 18,139 bp, and a pair of inverted repeats [IRs] of 26,070 bp. The genome contains 137 genes, including 89 protein-coding genes, 40 tRNA genes, and 8 rRNA genes. The GC contents in chloroplast genome, LSC region, SSC region, and IR region were 38.0%, 36.2%, 32.4%, and 42.9%, respectively. The phylogenetic analysis based on the plastid genomes showed that *A. tsinglingense* was more closely related with the congeneric *A. laevigatum*, *A. palmatum*, *A. wilsonii*, and *A. buergerianum*, these species were clustered into a monophyletic clade with high bootstrap support.

*Acer tsinglingense* W. P. Fang & C. C. Hsieh is an ecologically and economically important tree species in China. This species is mainly distributed in the mountain areas in western China. Previous studies of this species have mainly focused on the external morphological characters [Xu et al. 2008]. In this study, we characterized the complete plastid genome sequence of *A. tsinglingense* based on the Illumina pair-end sequencing data. The annotated plastid genome of *A. tsinglingense* has been deposited into the GenBank with the accession number MN393475.

The fresh and healthy leaves of *A. tsinglingense* were sampled in the Taiping National Forest Park (Xi’an, China; N 33.92346382, E108.65643740; Alt.941.41m). The voucher specimen was deposited at Northwest University Herbarium (LZH-2019-22). Total genomic DNA was isolated using the improved CTAB method (Doyle and Doyle 1987). Then, the DNAs were subjected to Illumina sample preparation, and pair-read sequencing was indexed by the Illumina Hiseq 2500 platform (San Diego, CA). In total, all raw reads were trimmed using the program NGSQCToolkit_version 2.3.3 (Patel and Jain 2012). After dislodged the low quality reads, the clean reads were assembled using MIRA version 4.0.2 (Chevreux et al. 2004), and MITObim version 1.8 (Hahn et al. 2013) using the plastid genome of *A. truncatum* (NC_037211) as the reference sequence. Annotation of plastid genome was conducted using the online program Dual Organellar Genome Annotator (DOGMA, Wyman et al. 2004), and then manually adjusted the positions of start codes and stop codes.

The complete plastid genome size of *A. tsinglingense* is 156,039 bp in length, including a large single-copy (LSC) region of 85,760 bp, a small single-copy (SSC) region of 18,139 bp, and a pair of inverted repeats (IRs) of 26,070 bp. The genome contains 137 genes, including 89 protein-coding genes, 40 tRNA genes, and 8 rRNA genes. The GC contents in plastid genome, LSC region, SSC region, and IR region were 38.0%, 36.2%, 32.4%, and 42.9%, respectively. A total of 14 genes (*tRNA-Lys* (UUU), *trnG tRNA*, *tRNA-Leu* (UAA), *tRNA-Val* (UAC), *tRNA-Ile* (GAU), *tRNA-Ala* (UGC), *rps16*, *atpF*, *rpoC1*, *petB*, *petD*, *rpl16*, *rpl2*, and *ndhB*) contained one intron, and three genes (*ycf3*, *clpP*, and *rps12*) contained two introns.

A total of 14 species from the genus *Acer* and *Dipteronia* were used to construct the phylogenetic tree with two *Euonymus* species as outgroups. All of the 16 plastid sequences were aligned using the software MAFFT (Katoh and Standley 2013) with the default parameters. The phylogenetic analysis was conducted using the program RAxML (Stamatakis 2006) with 1000 bootstrap replicates (Figure 1). The results showed that *A. tsinglingense* was more closely related with the congeneric *A. laevigatum*, *A. palmatum*, *A. wilsonii*, and *A. buergerianum*, these species were clustered into a monophyletic clade with high bootstrap support.

**Figure 1.**
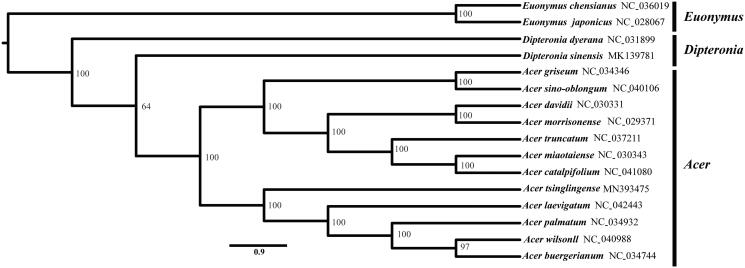
Phylogenetic relationship based on 16 complete plastid genomes.
